# Melanoma Cells Homing to the Brain: An *In Vitro* Model

**DOI:** 10.1155/2015/476069

**Published:** 2015-01-26

**Authors:** A. Rizzo, C. Vasco, V. Girgenti, V. Fugnanesi, C. Calatozzolo, A. Canazza, A. Salmaggi, L. Rivoltini, M. Morbin, E. Ciusani

**Affiliations:** ^1^Laboratory of Clinical Pathology and Medical Genetics, Foundation IRCCS Neurological Institute Carlo Besta, Via Celoria 11, 20133 Milano, Italy; ^2^U.O. Neuropathology, IRCCS Foundation C. Besta Neurological Institute, Foundation IRCCS Neurological Institute C. Besta, Via Celoria 11, 20133 Milano, Italy; ^3^Cellular Neurobiology Laboratory, Cerebrovascular Diseases Unit, Foundation IRCCS Neurological Institute C. Besta, Via Celoria 11, 20133 Milano, Italy; ^4^Neurologia-Stroke Unit, Manzoni Hospital, Via dell'Eremo 9/11, 23900 Lecco, Italy; ^5^Unit of Immunotherapy, Fondazione IRCCS National Cancer Institute, Via Venezian 1, 20133 Milan, Italy

## Abstract

We developed an *in vitro* contact through-feet blood brain barrier (BBB) model built using type IV collagen, rat astrocytes, and human umbilical vein endothelial cells (HUVECs) cocultured through Transwell porous polycarbonate membrane. The contact between astrocytes and HUVECs was demonstrated by electron microscopy: astrocytes endfeet pass through the 8.0 *μ*m pores inducing HUVECs to assume a cerebral phenotype. Using this model we evaluated transmigration of melanoma cells from two different patients (M1 and M2) selected among seven melanoma primary cultures. M2 cells showed a statistically significant higher capability to pass across the *in vitro* BBB model, compared to M1. Expression of adhesion molecules was evaluated by flow cytometry: a statistically significant increased expression of MCAM, *α*v*β*3, and CD49b was detected in M1. PCR array data showed that M2 had a higher expression of several matrix metalloproteinase proteins (MMPs) compared to M1. Specifically, data suggest that MMP2 and MMP9 could be directly involved in BBB permeability and that brain invasion by melanoma cells could be related to the overexpression of many MMPs. Future studies will be necessary to deepen the mechanisms of central nervous system invasion.

## 1. Introduction

Brain metastasis is the most common type of central nervous system (CNS) tumors being 10-fold more frequent compared to primary brain tumor [1, 2]. Cerebral metastasis appears in 30–40% of patients affected by systemic tumors [3] and the most common derive from lung cancer (17–39%), breast cancer (5–17%), and melanoma (8–11%) [4].

Melanoma has one of the highest CNS metastatic capability [5, 6] possibly due to the common embryological origin of melanocytes and neuronal subpopulations [7, 8]. Melanoma metastases have been shown to have a high mutation rate [9], variable phenotype, a clinically diffuse dissemination pattern [10], and the unique ability to elicit spontaneous antigen-specific host immune responses [11].

Brain is considered a privileged site for the presence of blood brain barrier (BBB) protecting it against the entry of toxic substances and microorganisms [12, 13]. Since the CNS also lacks the lymphatic system, the only way of metastatic cells to reach the brain is to cross this barrier [14, 15].

BBB is composed by endothelial cells (ECs), sheathing by astrocytic foot processes, and pericytes. Brain endothelial cells are anatomically different from the other endothelial cells and are characterized by the presence of tight junctions (TJ) and by the absence of fenestrations. Nevertheless the presence of BBB is not able to prevent parenchymal brain invasion from different tumor circulating cells and it is surprising that some cancer types give metastases preferentially to the brain [14]. This phenomenon draws the attention to the possibility that the BBB may have a supportive role in the formation of the metastasis as well: cerebral endothelial cells have been described to actively take part in the tumor cells transmigration process. Besides, giving protection to CNS-invading cells from the immune response and from chemotherapy, BBB may produce substances favorable for metastasis growth [16]. The vasculature of the BBB is unique because of its association with astrocytes: in the brain, astrocytes and endothelial cells are closely apposed, separated only by a thin basal lamina [17]; therefore, model involving physical contact between endothelial cells and astrocytes more closely represent the* in vivo* situation [18–20].

Adhesion and junction molecules involved in tumor cell-endothelial cell interaction are poorly known and even less is known about brain endothelial-specific mechanisms. Many authors suggest that tumor cells are able to partly mimic the molecular mechanisms of leukocyte-endothelial interaction occurring during inflammation. Depending on this, a role for integrins and adhesion molecules has been proposed to explain brain invasion [21, 22].

Indeed, the crossing of the BBB has been described to involve several mechanisms among which adhesion molecules, extracellular degradation molecules, and growth factors [14, 21–24]. Using an* in vitro* heterologous BBB model [14] in the present work we investigated which of these was predominant in melanoma to brain metastasization. Transmigration of melanoma cells from different patients was evaluated to identify and characterize cell cultures with different behaviour. Due to the features of the model, we focused our attention on adhesion [21–23] and extracellular matrix degradation processes [24].

## 2. Materials and Methods 

### 2.1. Cell Cultures

HUVECs primary cultures were isolated from healthy donors according to Jaffe method [25] and used in experiments up to the tenth passage. The typical purity of cells cultures was higher than 95% as assessed by flow cytometry after CD31 staining (polyclonal phycoerythrin-conjugated goat anti-human CD31, BD Bioscience). HUVECs were cultured in complete EBM (Endothelial Basal Medium, Lonza, Walkersville MD) medium completed with growth factors and antibiotics (EGM SingleQuots, Lonza) in cells culture dishes (Corning, USA) coated with Collagen S type I, 0.3 *μ*g/mL (Roche, Mannheim, Germany).

Rat astrocytes cells were commercially available primary cultures (rat brain cortex, Cambrex lot number 151104AT2) and were used in experiments up to the tenth passage. Cells were cultured in 75 cm^2^ flasks (Corning) in DMEM (Dulbecco's Modified Eagle's Medium, Gibco) completed with 10% of foetal bovine serum (FBS, Gibco) and antibiotics penicillin/streptomycin 1% (P/S, Gibco). Purity of cells cultures was tested by flow cytometry using an alexa fluor-conjugate antiglial fibrillary acid protein (GFAP) antibody; cells were used in experiments when the percentage of GFAP reached at least 95%.

Primary cultures of melanoma metastases were developed from surgical specimens as previously described [26] and were kindly provided by Dr. Licia Rivoltini (National Cancer Institute of Milan). Cells were cultured in 75 cm^2^ flasks (Corning) in RPMI (Gibco), in addition to10% FBS (Gibco) and P/S 1% (Gibco).

### 2.2. Establishment of the* In Vitro* BBB Model

*In vitro* BBB model was built with rat astrocytes and HUVECs cocultured through Transwell porous polycarbonate 8 *μ*m membrane (inner well), in 24 well plates (outer well) (Corning). The day before the preparation of the BBB model, the polycarbonate membrane was coated with 100 *μ*L of collagen IV at a concentration of 33 *μ*g/mL (mouse collagen, IV, BD Biosciences) for 1 h at room temperature (RT), then washed three times with phosphate buffered saline (PBS) without CaCl_2_ and MgCl_2_ (Gibco), and incubated in complete medium (100 *μ*L in the inner well and 600 *μ*L in the outer well) for 24 hours at 37°C in humidified atmosphere with 5% CO_2_. Astrocytes (200.000 cells) were seeded on the bottom side of the polycarbonate membrane insert, upside-down in humidified chamber for 1 hour. After that, the Transwell insert was inverted in a 24 well cell culture cluster (Costar, Corning Incorporated) in DMEM complete medium and placed over night at 37°C in humidified atmosphere with 5% CO_2_. After 24 hours, HUVECs (200.000 cells) were plated on the upper side of polycarbonate membrane and cultured in EBM complete medium. Astrocytes and HUVECs were than cocultured in EBM complete medium for four days before being used in transmigration experiments.

### 2.3. Trans-Endothelial Flux of L-Glucose

To assess the integrity of the* in vitro *BBB model, permeability of the barrier to L-glucose was tested as previously described [27]. Briefly, a solution of PBS with HEPES 20 mM containing [^14^C]-L-glucose 1 *μ*M (0.2 *μ*Ci/*μ*L, Amersham Biosciences) was prepared. Medium was removed from both inner and outer well of the Transwell and the outer well was filled with 600 *μ*L of PBS containing HEPES 20 mM, while the inner well was filled with 100 *μ*L of the solution containing [^14^C]-L-glucose. 10 *μ*L aliquots of the inner and outer well solution were removed at 10 minutes intervals for 1 hours, spotted onto glass fibre filters (Filtermat A, Wallac) and air-dried for 2 hours at room temperature. Glass fibre filters were than absorbed in liquid scintillation (Beta Plate Scint 1250-440) and counted (Trilux 1450 MicroBeta, Wallac).

### 2.4. Immunocytochemistry

After four days of coculture, Transwell inserts with HUVECs and astrocytes were fixed for immunocytochemical evaluation of representative markers of the BBB tight junctions, zonula occludens-1 (ZO-1) and vinculin. For ZO-1 detection inserts were fixed with a solution of paraformaldehyde-lysine-periodate (PLP) [28] in 0.1 M phosphate buffer, pH 7.4 (PB) for 20 min, while for vinculin detection paraformaldehyde 4% in PBS was used. After washes with PBS, a pretreatment for antigen unmasking was performed with a solution of 0.5% Triton X-100 in 50 mM MES pH 6, 3 mM EGTA, and 5 mM MgCl_2_, for 5 min, followed by washes with PBS and incubation with PBS containing 1% bovine serum albumin for 30 min. The following primary antibodies were incubated overnight at 4°C: ZO-1 (1 : 400, Zymed Lab., San Francisco, CA) and vinculin (1 : 400, Sigma, St. Louis, MO). In order to distinguish endothelial cells from astrocytes in the insert, double staining with biotinylated lectin from* Lycopersicon esculentum* (bLEA, Sigma Aldrich, St. Luis, MO), specific for endothelial cell glycocalyx residui (N-acetyl-D-glucosamine oligomers) [29, 30] was carried out. After washes with PBS, secondary antibodies were incubated for 1 hour at room temperature: for ZO-1 goat anti-rabbit DyLight TM 488 labeled (1 : 200, KPL, Gaithersburg, USA), for vinculin goat anti-mouse DyLight TM 488 labeled (1 : 200, Thermo Scientific, Rockford, USA), for bLEA Streptavidin Alexa Fluor 594 conjugated (1 : 200, Life Technologies, Eugene, USA). Negative controls, without the primary antibody, were also stained each run. Finally, nuclei were counterstained using DAPI 100 *μ*g/mL (Sigma-Aldrich) and a solution of PBS and Glycerol (1 : 2, Merck, Darmstadt, Germany) was added. Inserts were observed and analysed by Nikon D Eclipse C1 confocal laser-scanning microscope (Nikon, Tokyo, Japan).

### 2.5. Electron Microscopy (EM)

We checked for the presence of two crucial ultrastructural markers: the presence of HUVECs and astrocytes as a single layer, and the presence of tight junctions (sites of apparent fusion between two adjacent cells formed by the two opposite membranes and characterized by electron-dense material). After four days of coculture, specific polycarbonate membranes were removed from the inserts and specimens were fixed in 2,5% EM grade glutaraldehyde (Fluka Chemie, AG Buchs CH) in 0.05 M phosphate buffer (PB) pH 7.4, postfixed in 1% osmium tetroxide (EMS, Fort Washington, PA, USA) in 0.05 M PB, dehydrated in graded acetone and embedded in Spurr epoxy resin (EMS, Fort Washington PA USA). Thin sections 1 *μ*m thick were counterstained with toluidine blue and observed under optic microscope. Ultrathin sections (500 Å thick) of selected areas including HUVECs/astrocytes monolayers were harvested on 200 mesh copper grids (EMS), stained with uranyl acetate and lead citrate, and viewed under an electron microscope (Zeiss EM 109, Germany).

### 2.6. Transmigration Assay

Subconfluent cultured melanoma cells were harvested, washed in PBS, and labelled with the fluorescent tracer 5-6-carboxyfluorescein diacetate, succinimidyl ester (5(6)-CFDA, SE; CFSE) following manufacturer's instruction (Invitrogen, Italy). After CFDA labelling, cells were washed with PBS (with CaCl_2_ e MgCl_2_), centrifuged, and resuspended in complete medium (RPMI 10% FBS) at a concentration of 100.000 cells/100 *μ*L. 100 *μ*L of this solution were added to the inner well of the Transwell in which the* in vitro* BBB was established as previously described and incubated at 37°C. After 24 hours, transmigrated cells were collected in the outer well of the Transwell and counted by fluorescence microscopy using a Fuchs-Rosenthal chamber. Data regarding transmigrated cells are expressed as percentage of the total number of melanoma cells used. As controls, in any transmigration experiment, migration of melanoma cells through the polycarbonate membrane alone was also included. To confirm the presence of the endothelial and astrocytic monolayer, in each experimental session, one polycarbonate insert used in a transmigration experiment was processed for ultrastructural evaluation. Specimens were treated and viewed as described above; melanoma cells were identified by the presence of melanosomes in their cytoplasms.

### 2.7. Expression of Adhesion Molecules

HUVECs and melanoma cells were analysed by flow cytometry to evaluate the expression of the following surface proteins: ICAM-1, ICAM-2, VCAM-1, MCAM, *α*v*β*3, CD-49b, CD56(NCAM), and CCR7. Cells were labelled with the following fluorescein-5-isothiocyanate- (FITC-) or phycoerythrin- (PE-) conjugated antibody: ICAM-1/PE (Caltag Laboratories, cat. number 322708), ICAM-2/FITC (Alexis Corporation), VCAM-1/PE (BD Pharmingen, cat. number 555647), MCAM/PE (Beckman Coulter cat. number PN A07483), *α*v*β*3/FITC (Millipore, cat. number MAB1976F), CD49b/PE (eBioscience, cat. number 11-0498-41), CD56(NCAM)/FITC (cat. number 345811 BD Biosciences), and CCR7/FITC (eBioscience cat. number 11-1979-71).

Briefly, subconfluent cells were harvested with trypsin/EDTA (Gibco) and incubated with 1 *μ*g of each antibody at 4°C for 1 hour. Cells were washed ones with PBS, acquired using a FacsVantage SE (Becton Dickinson, CA, USA) and analysed by CellQuest Pro software (Becton Dickinson). Data are expressed as ratio between the mean fluorescence intensity (MFI) of the specific antibody and the MFI of the relative isotypic control (Simultest, *γ*1/*γ*2a, Becton Dikinson, USA). MFI ratio values greater than 1 indicate expression of the molecule on the cell surface.

### 2.8. Real Time PCR

Total RNA was isolated by RNeasy Mini Kit (Qiagen, Milano, Italy) following manufacturer's instructions and quantified by NanoDrop 2000 Spectrophotometer (Thermo Scientific, Wilmington, USA). Equal amounts of RNA of each sample were reverse transcribed using RT^2^ First Strand Kit (Qiagen, Italy). The expression of 84 different tumor-related genes was conducted by Human Tumor Metastasis RT^2^ Profiler PCR Array (Qiagen, Italy) using RT^2^ SYBR Green ROX qPCR Master Mix and 100 ng of total RNA for each reaction, following the manufacturer's protocol. Ct values obtained were normalised to the Ct value of *β*-actin, the most conserved housekeeping gene present in the array (ΔCt) and ΔΔCt was calculate as ΔCt_M2_ − ΔCt_M1_. Data are expressed as fold change (2^−ΔΔCt^) in M2 compared to M1. To validate the results of the tumor metastasis array, changes in the expression of selected genes (MCAM, PLAUR, VEGFA, SYK, and HPSE) was confirmed by single Taqman assays (Applied Biosystems, Germany) on two different biological replicates.

## 3. Results

### 3.1. *In Vitro* BBB Model

**Figure 1:**
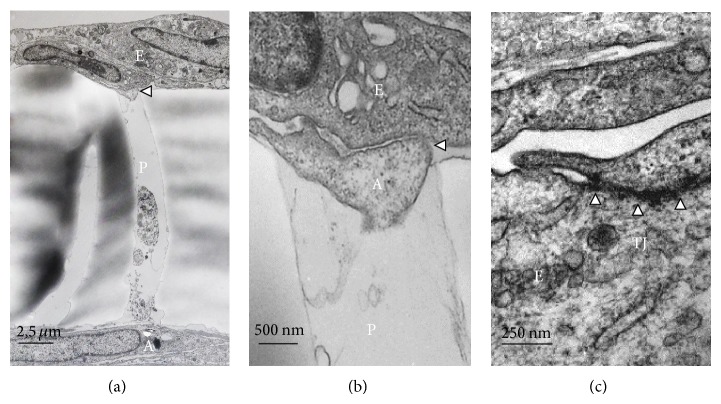
(a) The endothelial cells (E) were plated on the upper side of polycarbonate membrane at a density of 2 · 10^5^/well and the astrocytes (A) were cultured on the bottom side of the membrane at a density of 2 · 10^5^/well. The arrowhead shows the contact between astrocyte endfeet and endothelial cells monolayer across a pore (P) of the membrane. (b) Contact (arrowhead) between astrocyte endfeet (A) and endothelial cells (E) across a pore (P) at higher magnification. (c) Transversal section of endothelial cells monolayer (E) after four days of cocultures with astrocytes (see Material and Methods). Arrows indicate the tight junction (TJ) between two adjacent endothelial cells.

We developed an* in vitro* BBB model which is a “contact through-feet” model. It is built with human endothelial cells deriving from HUVECs and rat astrocytes cocultured in Transwell 8.0 *μ*m porous polycarbonate membrane (see Materials and Methods for further details). We obtained a model that satisfied some of the* in vivo* BBB characteristics; by electron microscopy we demonstrated that our model allowed astrocytes endfeet to physically contact HUVECs through membrane pores (Figure 1(a)) inducing the formation of tight junctions (Figure 1(b)) which is a morphological feature indicating the acquisition of functional properties of the cerebral endothelium.

**Figure 2:**
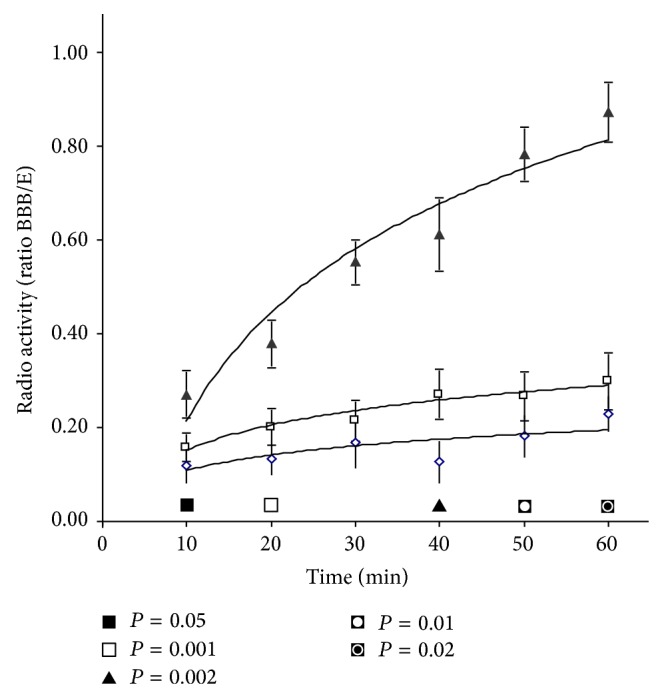
Graphic representation of [^14^C]L-glucose permeability assay in our* in vitro* BBB model (see Material and Methods). A statistically significant decrease to L-glucose permeability was detected in cocultures after four days (-◊-) if compared to polycarbonate membrane alone (-▲-) or to HUVEC alone (-□-) plated on the upper side of the polycarbonate membrane for four days. [^14^C]L-Glucose permeability was expressed as ratio between the radioactivity measured in the lower chamber and the radioactivity measured in the upper chamber of the Transwell. A ratio of 1 means complete equilibrium between the upper and the lower well of the Transwell. Data are expressed as mean values ± SD (error bars) from at least three independent experiments. Statistics:* in vitro* BBB versus HUVEC alone (*t*-test).

To investigate the “tightness” of this model, we evaluated the permeability of our* in vitro *BBB to [^14^C]-L-glucose [27]. We measured radioactivity content in the medium of HUVECs cocultured with astrocytes and of HUVECs or polycarbonate inserts alone sampling medium at 10 minutes intervals for 1 hour. The ratio between radioactivity measured in the lower chamber and the radioactivity measured in the upper chamber of the Transwell showed that L-glucose permeability was strongly reduced by the presence of the monolayer of HUVECs alone and even more reduced when HUVECs were cocultured with astrocytes (Figure 2). The comparison between* in vitro* BBB and the HUVECs monolayer alone showed a statistically significant reduction in permeability as early as 10 minutes after the addition of [^14^C]-L-glucose in the upper chamber. In the insert with polycarbonate membrane alone, the equilibrium was almost reached after 60 minutes (Figure 2).

**Figure 3:**
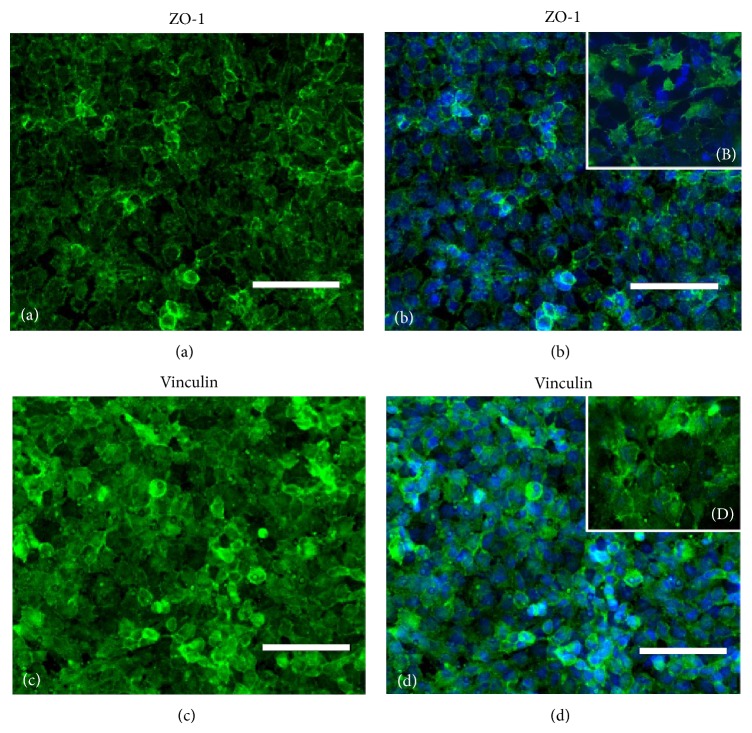
The panel shows the localization of two markers representative of the blood brain barrier tight junctions. (a) Expression of zonula occludens-1 (ZO-1) focalized in membrane producing a linear staining at cell boundaries and (b) merge of ZO-1 and nuclear staining (10x magnification); (B) magnification detail of ZO-1 and DAPI staining (40x magnification); (c) membrane-cytoskeletal protein of focal adhesion plaques vinculin protein; and (d) merge of vinculin and nuclear staining (10x magnification); (D) magnification detail of vinculin and DAPI staining (40x magnification). DAPI: blue; ZO-1: green; vinculin: green. Scale bar: 100 *μ*m.

The model was further characterised analysing the expression of zonula occludens-1(ZO-1) and vinculin (Figure 3). The immunoreactivity for ZO-1 was localized at the boundaries of endothelial cells. Labelling for vinculin showed a slightly different pattern, mainly distributed as a diffuse cytoplasmic band at endothelial cell periphery and around the nucleus.

### 3.2. Transmigration Assay

The ability of 7 metastatic melanoma primary cultures to transmigrate across our* in vitro* barrier model was screened in preliminary experiments to estimate if they had different ability to transpass the BBB model (data not shown).

**Figure 4:**
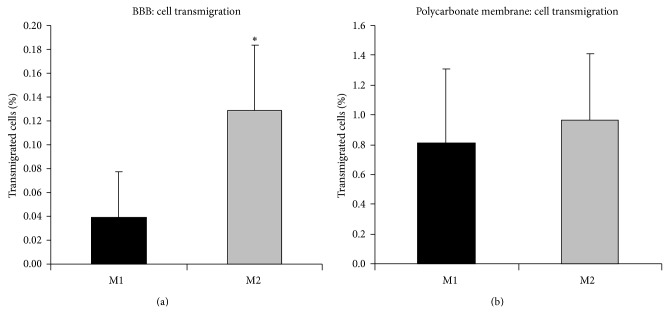
(a) Transmigration of M1 and M2 cells across the HUVECs monolayer cocultered with astrocytes (*in vitro* BBB). 100.000 cells were added in the upper chamber and, after 24 hours, transmigrated cells were counted in the lower chamber (see Material end Methods). Transmigrated cells are expressed as percentage of the total number of melanoma cells used for each insert. Data are expressed as mean ± SD (error bars) from at least six independent experiments. M2 cells showed a statistically significant increased migration compared to M1 (^*^ *P* = 0.000016,* t*-test). (b) Transmigration ability through the polycarbonate membrane alone was similar in M1 and M2 cells. 100.000 cells were loaded in the upper well of the Transwell chamber and incubated for 24 h. Data are expressed as mean percentage of the total number of cells ± SD (error bars) from at least six independent experiments.

Among the various primary cultures analyzed, two (named M1 and M2) were selected for more detailed analysis based on their different capability to transmigrate across the* in vitro* BBB model. The M2 cell culture showed a statistically significant higher capability to cross the* in vitro* BBB model when compared to M1 (Figure 4(a)). As a matter of fact, M1 showed a mean percentage of migrated cells of 0.038% while M2 showed a mean value of 0.13% (*P* = 0.000016, Student's *t*-test).

To exclude major differences in generic motility of the two primary cultures in our experimental model, transmigration ability across the polycarbonate membrane alone was also measured and no differences were detected (Figure 4(b)). These data suggest that the presence of the* in vitro* BBB is actually responsible of the observed differences in cells transmigration among the two cell lines.

**Figure 5:**
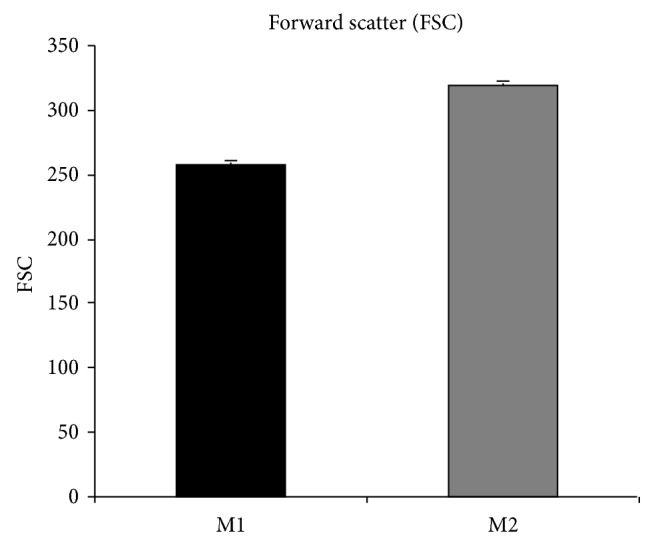
To exclude major differences in cell volume between M1 and M2 cells eventually accounting for the different ability to transmigrate, the forward scatter of M1 (black bar) and M2 (grey bar) cell was measured by flow cytometry. Despite M2 showing a statistically significant increased migration through the* in vitro* BBB compared to M1, their volume was slightly higher than that of M1 (310 versus 250 arbitrary unit) suggesting that cell volume was not responsible for the differences observed in transmigration.

Since the experimental model forces cells to pass through 8 *μ*m pores, we assumed the cell volume as a relevant parameter in our assay. We therefore measured the cellular volume using flow cytometry; forward scatter was measured for both cell lines and results are reported in Figure 5. No statistically significant differences were detected in cellular volume between M1 and M2 cells.

### 3.3. Expression of Metastasis-Related Molecules

**Figure 6:**
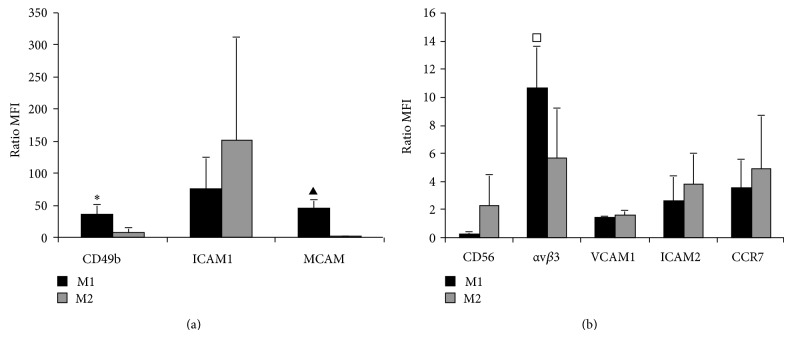
Surface adhesion molecules expression in M1 (black bars) and M2 cells (grey bars). Cultured cells were stained with the fluorochrome-conjugated specific antibody for the listed molecules and analyzed by flow cytometry (see Material and Method). Data are expressed as means ± SD (error bars) of the ratio between the mean fluorescence intensity (MFI) of specific antibody and the MFI of the relative isotypic control. Values greater than 1 indicate expression of the marker (see Material and Methods). Data refers to at least three independent experiments. Statistics: M1 versus M2 (*t*-test): ^□^ *P* = 0.019; ^*^ *P* = 0.0003; ^▲^ *P* = 0.00005.

To investigate putative molecular mechanisms responsible for the different transmigration behaviour observed in M1 and M2, we analyzed the expression of some proteins potentially involved in adhesion and transmigration process like integrins and adhesion molecules by cytofluorometry: CD49b, ICAM1, MCAM, CD56, *α*v*β*3, VCAM1, ICAM2, and CCR7 were analysed and the data obtained are summarized in Figure 5. A statistically different expression was detected for *α*v*β*3, CD49b, and MCAM, which were more expressed in M1 compared to M2 cells (Figures 6(a) and 6(b); *P* < 0.05, Student's *t*-test). On the other hand, a trend to decreased expression of ICAM-1 and CD56 was detected in M1 cells even if no statistically significant differences were found (Figure 6(a)).

To identify differentially expressed genes possibly contributing to the increased ability of M2 cells to cross the* in vitro* BBB, mRNA expression of 84 genes involved in tumor metastasization was screened using a specific real time PCR-based array that allowed amplification of different groups of transcripts (see Material and Methods). The array enabled us to analyze different tumor metastasis-related genes involved in cell adhesion, cell cycle, cell growth and proliferation, apoptosis, extracellular matrix protein, transcription factors, and regulators.

Data obtained underwent statistical evaluation and only genes that were expressed less or more than twofold (2^−ΔΔCt^) were considered, respectively, downregulated or upregulated in the M2 cells which displayed the highest transmigration ability in our* in vitro* model. A complete list of the results obtained is reported in Supplementary Table 1 (see Supplementary Material available online at http://dx.doi.org/10.1155/2015/476069). Since migration of melanoma cells was measured after 24 hours, we assumed as marginal the effects of proliferation in our experimental conditions and in Tables 1(a) and 1(b) are summarised the results concerning regulation of adhesion molecules (AM) and extracellular degradation molecules (EMD) genes.

### 3.4. Downregulated Genes

Ten out of the 14 downregulated genes (71.5%) encoded for adhesion molecules (AM) while 28.5% encoded for extracellular matrix degradation molecules (EMD). SYK, a tyrosine kinase widely expressed in hematopoietic cells, displayed the highest decrease (−91.72) followed by melanoma cell adhesion molecule (MCAM) (Table 1(a)).

### 3.5. Upregulated Genes

Genes encoding for molecules involved in cell growth/proliferation (CG/P) were the most represented in the group of upregulated genes (54.8% see supplementary Table 1); however, genes involved in the synthesis of adhesion molecules and matrix degradation molecules are shown in Table 1(b).

## 4. Discussion

CNS metastasis is rather common among patients with melanoma and there is a growing interest in the development of* in vitro* BBB models to better understand the molecular mechanism of invasion of the CNS by malignant tumor cells.

Several previous studies have underlined the importance of both astrocytes and endothelial cells to reproduce* in vitro* structural and functional characteristics of the BBB* in vivo* [16, 31–36]. Since glial factors are well conserved among species, many heterologous models have been described so far [37–40].

The pivotal role of astrocytes in maintaining cerebral homeostasis, participating in neural signal transduction and transporting nutrients from the circulation to the neurons and buffering ionic imbalance, has been underlined by a number of authors [41–44]; in the present study we developed an* in vitro* contact through-feet BBB model built with human noncerebral endothelial cells and rat astrocytes.

The isolation of human cerebral endothelial cells is still a rare option; therefore* in vitro* models have been often developed with extracerebral human endothelial cells forced to assume a cerebral phenotype by the contact with astrocytic endfeet or by astrocyte-conditioned culture media [18, 20, 45–47]. In our experimental conditions, the ability of astrocytic endfeet to take contact with HUVECs is clearly demonstrated by electron microscopy. Indeed, the HUVECs monolayer displayed tight junctions further supporting the idea that in coculture models noncerebral endothelial cells may be induced to assume features typical of the cerebral endothelium [18–20, 20–22]. The tightness of our* in vitro* BBB model was further supported by the significant decrease in [^14^C]-L-glucose permeability detected when both HUVECs and astrocytes were in coculture, in agreement with previously reported data about permeability of the BBB to [^14^C]-L-glucose, sucrose, or drugs [27, 48–50].

Transmigration data showed that in our model the percentage of cells crossing the* in vitro* BBB model was less than 0.2%. This is in line with previously published data in similar models [51, 52]. As a further control, we have tested the migration of A375 (a commercially available melanoma cell line) in our model and we found similar transmigration ability if compared to what was previously reported by other authors for the same cell line in a similar* in vitro* model (data not shown) [53]. However, also* in vivo* studies have shown that less than 0.1% of the cells survive more than 24 h after injection contributing to the concept that metastasization is an inefficient process [54].

In our experimental conditions, M1 and M2 cell migration was similar when tested through the sole polycarbonate membrane and was statistically different only if HUVECs and astrocytes where present in the model (Figure 3). Taken together, these findings suggest that the different behaviour of the two cell cultures was induced by the presence of the barrier, further supporting that* in vitro* BBB models might be useful to study CNS-specific migration.

Since the mean volume of M1 and M2 cells was similar, it seems that in our model the cell size was not a limiting event in transmigration. This observation further suggests that the different migratory behaviour depended on the* in vitro* barrier described rather than intrinsic differences between the two cell lines. However, arrest of tumour cells* in vivo* was found to take place at the level of capillaries where the diameter of the vessels is similar to those of the metastatic cells suggesting a key role for the cell size in early phases of metastasis formation [55, 56]. Although* in vitro* models might be used in the study of the molecular mechanisms involved in the crossing of the BBB, they can hardly be used in elucidating all the complex phases of the metastatic process [14].

Surprisingly, our findings showed a general decrease in the expression of adhesion molecules in the melanoma primary cell culture displaying the higher* in vitro* migration rate. In fact, this decrease was significant for CD49b, NCAM, and alfaVbeta3 integrin in flow cytometry experiments and was confirmed by the reduced level of expression detected by real time PCR of these and of other adhesion molecules (Table 1). The expression of beta3 integrin has been recently analyzed by Berghoff el al. [57] in a study on autopsy specimens which also included melanoma metastasis; beta3 integrin score was reported to be generally low in all metastasis and very low or absent in melanoma metastasis. Our observations are in agreement with these data and would support a negative role for beta3 integrin in CNS metastatization.

On the other hand, in contrast with several evidences supporting a role for MCAM overexpression in metastatic transformation of various types of tumors including melanoma [58] prostate cancer [59] and non-small-cell lung carcinoma [60], our data would suggest that a reduced expression of MCAM promotes migration. This hypothesis is also in contrast with previously published data which clearly showed a role for MCAM in cell motility in melanoma [61]. However, in our experimental conditions, we could not detect a statistically significant difference in the migration of the two cell lines through the sole polycarbonate membrane. Since MCAM was expressed on both cell lines, albeit at much lower level in M2, its role in our model might be marginal.

The increased ability to cross the* in vitro* BBB detected in M2 cells compared to M1 correlated with the higher expression of some of MMPs in this cell line. MMPs are primarily involved in extra cellular matrix degradation; however, the literature data also showed their complex role in creating and maintaining the appropriate microenvironment for tumor growth at primary and metastatic sites [62]. A role for MMPs in BBB disruption has been previously reported; indeed, Fazakas et al. showed that melanoma cells can metastasize to the brain disrupting the continuity of tight junctions by producing proteases and that protease protease inhibitor might significantly reduce the number of extravasating melanoma cells [63]. Although in a different cellular model, our results are in agreement with previously reported data showing that MMP2 and MMP9 are directly involved in TJs disruption [64–66]. Moreover they suggest that other MMPs (MMP7, MMP10, MMP11, and MMP13) might be implicated in CNS invasion by metastatic melanoma cells.

Due to the restricted accessibility to the CNS to chemotherapeutics, the prevention of the formation of a brain metastasis is of crucial importance; therefore the understanding of the mechanism involved in this complex process is a key step in developing new therapeutic options.

## Supplementary Material

Supplementary table 1 shows a complete list of the differentially expressed genes in M1 and M2 cells as obtained by the metastasis specific Real time PCR-based array (see materials and methods, section 2). Data are expressed as fold decrease (a) or increase (b) in M2 compared to M1.

## Figures and Tables

**Table 1:** Differentially expressed genes in M1 and M2 cells as obtained by the metastasis specific real time PCR-based array (see materials and methods, Section 2). Data are expressed as fold decrease (a) or increase (b) in M2 compared to M1.

Symbol	Gene name	Fold	Function
(a)			
SYK	Spleen tyrosine kinase	−91.72	AM
MCAM	Melanoma cell adhesion molecule	−29.76	AM
VEGFA	Vascular endothelial growth factor A	−7.85	AM
CTNNA1	Catenin (cadherin-associated protein), alpha 1	−6.51	AM
ITGB3	Integrin, beta 3	−6.46	AM
FAT1	FAT atypical cadherin 1	−5.36	AM
HPSE	Heparanase	−5.05	EMD
CD44	CD44 molecule	−5.05	AM
TIMP2	Metallopeptidase inhibitor 2	−4.72	EMD
COL4A2	Collagen, type IV, alpha 2	−4.60	EMD
APC	Adenomatous polyposis coli	−4.02	AM
PNN pinin	Desmosome associated protein	−3.53	AM
MMP3	Matrix metallopeptidase 3 (stromelysin 1, progelatinase)	−2.58	EMD
MTSS1	Metastasis suppressor 1	−2.57	AM

(b)			
CDH1	Cadherin 1, type 1, E-cadherin	533	AM
MMP2	Matrix metallopeptidase 2 (gelatinase A, 72 kDa gelatinase, 72 kDa type IV collagenase)	54.35	EMD
ITGA7	Integrin, alpha 7	15.54	AM
CDH6	Cadherin 6, type 2, K-cadherin (fetal kidney)	8.23	AM
MMP10	Matrix metallopeptidase 10 (stromelysin 2)	6.83	EMD
TIMP3	TIMP metallopeptidase inhibitor 3	4.92	EMD
CDH11	Cadherin 11, type 2, OB-cadherin (osteoblast)	3.97	AM
MMP9	matrix metallopeptidase 9 (gelatinase B, 92 kDa gelatinase, 92 kDa type IV collagenase)	3.58	EMD
MMP11	Matrix metallopeptidase 11 (stromelysin 3)	3.55	EMD
MMP13	Matrix metallopeptidase 13 (collagenase 3)	3.31	EMD
MMP7	Matrix metallopeptidase 7 (matrilysin, uterine)	2.61	EMD
MGAT5	Mannosyl (alpha-1,6-)-glycoprotein beta-1,6-N-acetylglucosaminyltransferase	2.22	AM
FN1	Fibronectin 1	2.08	AM
CTSL1	Cathepsin L	2.06	EMD
